# Chemical Characterization and In Vitro Antioxidant, Anti-Inflammatory, and Colon Cancer-Preventive Potential of a Polysaccharide Fraction from *Macrolepiota procera*

**DOI:** 10.3390/ijms26209978

**Published:** 2025-10-14

**Authors:** Natalia Nowacka-Jechalke, Marta Kinga Lemieszek

**Affiliations:** 1 Department of Pharmaceutical Botany, Medical University of Lublin, Chodźki 1, 20-093 Lublin, Poland; 2 Department of Medical Biology, Institute of Rural Health, Jaczewskiego 2, 20-090 Lublin, Poland; martalemieszek@gmail.com

**Keywords:** parasol mushroom, β-glucans, anticancer potential, antiproliferative effect, cytotoxicity, antioxidants

## Abstract

Polysaccharides from edible mushrooms are increasingly recognized as bioactive compounds with health-promoting properties. In this study, a polysaccharide-rich fraction Mp-CPS was isolated from fruiting bodies of *Macrolepiota procera* using ultrasound-assisted extraction. The chemical composition of crude polysaccharides from the parasol mushroom was evaluated using spectrophotometric and electrophoretic methods. Chemical analysis revealed that Mp-CPS is mainly composed of glucose- and galactose-based heteropolysaccharides, with β-glucans as the predominant glucan type. The biological potential of Mp-CPS was evaluated in light of its antioxidant, anti-inflammatory, and anticancer activities. Antioxidant assays (TEAC, ORAC) demonstrated significant radical-scavenging capacity, with higher activity observed in the ORAC test. As revealed by biochemical examination, Mp-CPS also inhibited key pro-inflammatory enzymes: COX-1, COX-2, and LOX. At the same time, in vitro research (MTT and LDH assays) has shown the great chemopreventive abilities of Mp-CPS against human colon cancer cells, which intensified with the degree of cell malignancy. Overall, these results highlight *M. procera* as a sustainable and valuable source of biologically active polysaccharides with antioxidant, anti-inflammatory, and anticancer potential. The findings support further exploration of Mp-CPS for applications in functional foods and nutraceuticals.

## 1. Introduction

Mushroom-derived polysaccharides have attracted considerable scientific interest due to their broad spectrum of health-promoting properties and relatively low toxicity. These macromolecules are typically high-molecular-weight carbohydrates composed of monosaccharide units linked by glycosidic bonds, often forming complex branched or linear structures. They include β-glucans, α-glucans, heteropolysaccharides, and glycoprotein complexes, with β-(1→3)- and β-(1→6)-linked glucans being the most extensively studied [1]. The biological activity of these polysaccharides is closely related to their structural characteristics, such as monosaccharide composition, glycosidic linkage type, branching degree, molecular weight, and conformational features (e.g., triple-helical arrangements). Variations in these parameters influence their solubility, receptor-binding affinity, and subsequent modulation of cellular pathways [2]. Functionally, mushroom polysaccharides have been reported to exert immunomodulatory, antioxidant, anti-inflammatory, prebiotic, and anticancer effects [3,4,5]. They can activate innate and adaptive immune responses, stimulate the production of cytokines, and enhance immune cell activity, thereby contributing to the prevention and management of chronic diseases, including cancer [4]. Mushroom polysaccharides act as biological response modifiers (BRMs) by stimulating macrophages, dendritic cells, natural killer (NK) cells, and T lymphocytes, which enhances immune surveillance and contributes to the inhibition of tumor initiation, promotion, and progression [6]. Their low toxicity, combined with pleiotropic bioactivities, makes them promising candidates for adjuvant therapy in cancer prevention and treatment. Current studies also indicate their ability to complement conventional therapies, reduce chemotherapy-induced immunosuppression, and improve the overall efficacy of anticancer regimens [7]. Mushroom-derived polysaccharides possess unique chemical compositions and structures, reflected in their broad spectrum of bioactivities. Unlike proteins or nucleic acids, monosaccharides can form multiple glycosidic linkages, giving rise to a wide array of linear and branched architectures. This versatility makes polysaccharides among the most structurally complex biopolymers, with significant potential to modulate various biological processes [8]. It should be emphasized that the extraction strategy is a key determinant of their structural integrity and functional properties. Conventional methods, such as hot water or acid/alkali extraction, often involve prolonged heating, which can compromise stability and require extensive purification. In contrast, advanced extraction technologies—such as ultrasound-assisted extraction (UAE), microwave-assisted extraction (MAE), enzyme-assisted extraction, and subcritical water extraction—reduce thermal and mechanical stress, improve yield, and better preserve bioactive properties [9]. Therefore, it is imperative to intensify research on the optimization of extraction conditions, structural diversity, mechanisms of action, and therapeutic applications of mushroom polysaccharides, as such investigations hold significant potential for the development of safe and effective strategies for the prevention and treatment of chronic diseases and cancer.

*Macrolepiota procera* (Scop.) Singer, commonly known as the parasol mushroom, is a widely distributed edible species in Europe, including Poland, and is appreciated for its delicate, nutty flavor and firm texture. Mature fruiting bodies are readily identified by their tall, slender stipe, large umbrella-like cap with brown scales, and lamellar hymenophore [10]. This species inhabits forests, meadows, and clearings from late summer to autumn, and methods for its cultivation have also been described [11]. Nutritionally, *M. procera* is low in calories and fat yet rich in dietary fiber, carbohydrates, high-quality proteins, minerals, and vitamins [10]. Beyond its nutritional value, it contains diverse bioactive compounds contributing to antibacterial [12], antioxidant [13], anti-inflammatory, and immunomodulatory [14] effects. Considering the biological activity of polysaccharides, it has so far been demonstrated that they exhibit marked antioxidant properties [5,15] and prebiotic potential [16]. Beyond in vitro assays, mycelial polysaccharides from *M. procera* have been shown to protect mice against nonylphenol-induced reproductive toxicity by alleviating oxidative stress, cell damage, and inflammation [17]. Furthermore, water-extractable polysaccharides from *M. procera* have been reported to stimulate immune responses in vitro, displaying notable immunomodulatory activity [14].

Anticancer properties of *M. procera* have been reported so far, though most available data concern organic solvent extracts. For instance, *M. procera* fruiting body extracts exhibited cytotoxic activity against human lung adenocarcinoma A549 cells [18]. Additionally, silver nanoparticles synthesized using *M. procera* extract demonstrated cytotoxicity against multiple human cancer cell lines, including MCF-7 (breast), A549 (lung), Saos-2 (bone), and HT-29 (colon) [19]. Despite the indicated anticancer effects of some extracts, studies strictly dedicated to polysaccharides—which are the main class of compounds responsible for such activities—remain limited [3,4,5,6]. Therefore, the present study, focused on evaluating the chemopreventive properties of *M. procera* polysaccharides, is novel and contributes valuable preliminary insights. Investigation of other biological activities of these compounds is also warranted, as knowledge in this area remains insufficient. The identification and characterization of *M. procera*-derived polysaccharides must be supported by detailed chemical analysis, given their structural diversity and the resulting variety of mechanisms of action. Considering the structural complexity and biological relevance of fungal polysaccharides, their efficient isolation is essential for in-depth study. Ultrasound-assisted extraction (UAE) has proven particularly effective for obtaining cell wall polysaccharides, enhancing yield while preserving native structure and bioactivity [1]. Highlighting UAE underscores its potential in facilitating the investigation of *M. procera* polysaccharides and their diverse biological activities.

Despite extensive research on mushroom-derived polysaccharides, many edible species, including *M. procera*, remain underexplored as natural sources of bioactive compounds. Based on this rationale, the present study aimed to isolate and chemically characterize crude polysaccharides from *M. procera* fruiting bodies and to evaluate their biological activities, including antioxidant properties, inhibition of pro-inflammatory enzymes, and, especially, direct anticancer potential against human colon cancer cell lines. By addressing these objectives, the study contributes to the identification of new natural sources of bioactive polysaccharides and supports the ongoing search for compounds with chemopreventive and therapeutic applications.

## 2. Results

### 2.1. Chemical Composition of Crude Polysaccharides from M. procera

The crude polysaccharide fraction from *M. procera* Mp-CPS was obtained using the previously optimized extraction procedure [20]. The applied method yielded a polysaccharide fraction with an extraction efficiency of approximately 15.7 ± 0.38%, confirming the effectiveness of the selected method.

The composition of the obtained crude polysaccharides was analyzed using spectrophotometric and electrophoretic methods, performing both qualitative and quantitative assessments of the major metabolite groups. Mp-CPS represents a water-soluble fraction consisting predominantly of sugars, which account for 63.86 ± 0.92% (Table 1). The contents of the remaining analyzed compound groups were considerably lower, with uronic acids at 6.71 ± 0.21%, proteins at 4.01 ± 0.18%, and polyphenolic compounds at only 2.19 ± 0.09% (Table 1). This indicates the effectiveness of the procedures applied during the extraction process in removing other constituents, allowing for the rapid and efficient isolation of a relatively pure polysaccharide fraction. Moreover, the glucan content in the fruiting bodies of *M. procera* was determined, and the results (Table 1) indicate that the majority of total glucans (13.51 ± 0.41 g/100 g d.w.) are β-glucans (10.76 ± 0.53 g/100 g d.w.), whereas α-glucans are present only in minor amounts (2.75 ± 0.13 g/100 g d.w.).

**Figure 1:**
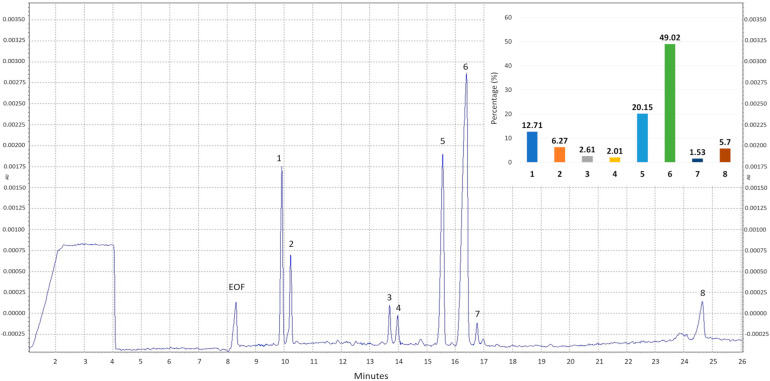
Electropherogram of the crude polysaccharides from *M. procera* Mp-CPS. EOF−electroosmotic flow; 1—mannitol; 2—trehalose; 3—unknown I; 4—fucose; 5—galactose; 6—glucose; 7—mannose; 8—unknown II and the bar chart of percentage distribution of monosaccharides in Mp-CPS. Migration times of standards: mannitol—8.8812; trehalose—9.1729; fucose—12.9198; galactose—14.4872; glucose—15.2462; mannose—15.7151.

The composition of the polysaccharide fraction isolated from the fruiting bodies of *M. procera* was qualitatively analyzed by high-performance capillary electrophoresis (HPCE) following prior hydrolysis of the sample with trifluoroacetic acid (TFA). The analysis revealed the presence of eight carbohydrate-derived peaks (Figure 1). Based on migration times, six of the eight compounds were identified, while two remained classified as unknown. The relative percentage distribution of the individual sugars was calculated from the peak areas and is presented in the bar chart in Figure 1.

Glucose was identified as the predominant sugar in Mp-CPS, accounting for nearly half of the total monosaccharides (49.02%). Galactose was the second most abundant sugar (20.15%), followed by mannitol (12.71%), a polyol derived from mannose, while mannose itself was also present in the sample but at the lowest level (1.53%). Trehalose, a disaccharide composed of two glucose units linked via an α,α-1,1-glycosidic bond was also detected (6.27%). Moreover, the analysis revealed the presence of two peaks from unidentified carbohydrates (Unknown I and Unknown II). Both of these compounds represent only minor components of the analyzed sample, accounting for 2.61% and 5.70%, respectively.

### 2.2. Biological Activity of Crude Polysaccharides from M. procera

#### 2.2.1. Antioxidant Activity

The antioxidant activity of polysaccharides from *M. procera* was evaluated using two complementary assays, TEAC and ORAC, which assess different mechanisms of radical scavenging and oxidative inhibition. Our study revealed that Mp-CPS exhibits measurable antioxidant properties. As shown in Table 2, the antioxidant capacity of Mp-CPS determined by the TEAC assay was 102.00 ± 2.09 µM Trolox/g of crude polysaccharides. Evaluation using the ORAC assay revealed a threefold higher activity, reaching 358.56 ± 13.18 µM Trolox/g of Mp-CPS.

#### 2.2.2. Anti-Inflammatory Potential

The next step focused on the anti-inflammatory activity of crude polysaccharides from *M. procera*. The effect of Mp-CPS on the activity of pro-inflammatory enzymes, including cyclooxygenase-1 (COX-1), cyclooxygenase-2 (COX-2), and lipoxygenase (LOX), was investigated. As shown in Table 3, Mp-CPS effectively reduced the activity of all enzymes tested. The most pronounced effect was observed for COX-1, with an inhibition of 74.23 ± 1.26%. Notably, Mp-CPS was almost twice as effective as acetylsalicylic acid (ASA), a commonly used non-steroidal anti-inflammatory drug, which inhibited COX-1 activity by 40.21 ± 0.78%. While Mp-CPS also inhibited LOX (43.69 ± 0.94%) and COX-2 (39.09 ± 0.42%), ASA demonstrated stronger inhibition for these enzymes (92.86 ± 0.51% and 96.84 ± 0.93%, respectively). These results indicate that Mp-CPS is a potent inhibitor of COX-1, while exhibiting moderate inhibitory activity against COX-2 and LOX. To the best of our knowledge, this is the first study examining the effect of polysaccharides isolated from *M. procera* on pro-inflammatory enzymes.

#### 2.2.3. Anticancer Potential

The anticancer potential of crude polysaccharides from *M. procera* was examined in an in vitro model of colon cancer, which is one of the most common types of cancer in the light of the incidence and mortality rate [21]. The study was conducted on three colon cancer cell lines which represent the successive stages of its development according to Dukes classification (Caco-2: Dukes B, LS180: Dukes B, HT-29: Dukes C). Simultaneously, Mp-CPS’s influence on human normal colon epithelial cells CCD 841 CoN was tested.

**Figure 2:**
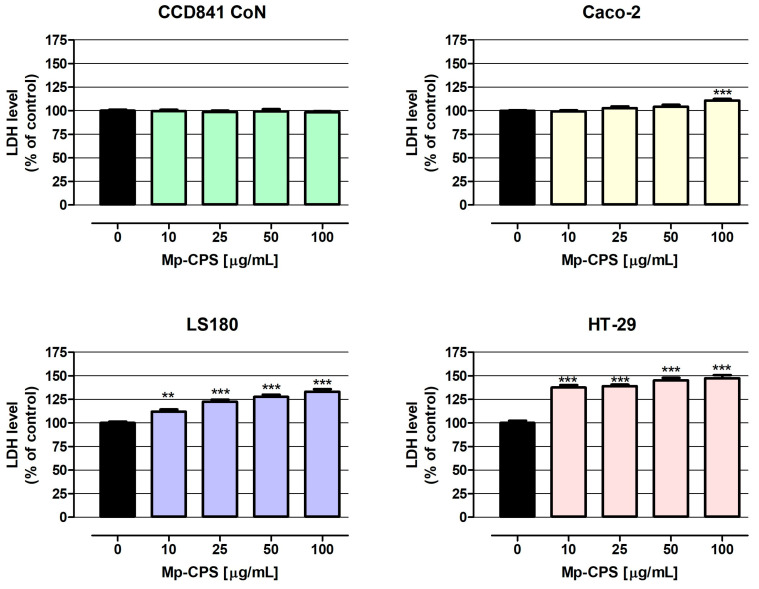
Cytotoxic influence of crude polysaccharides from *M. procera* on both normal and cancer colon cell lines. Human colon epithelial CCD841 CoN cells and human colon adenocarcinoma Caco-2, LS180, and HT-29 cells were treated with the crude polysaccharides Mp-CPS at concentrations of 10, 25, 50, and 100 μg/mL for 24 h. Mp-CPS cytotoxicity was examined photometrically by the LDH assay. Results are presented as mean ± SEM of at least 4 measurements. ** *p* < 0.01 versus control, *** *p* < 0.001 versus control, one-way ANOVA test; post-test: Dunnett.

The results of the LDH assay (Figure 2) have shown cytotoxicity in all investigated cancer cell lines in response to Mp-CPS. The most sensitive for tested polysaccharides were HT-29 cells, wherein Mp-CPS even at the lowest tested concentration (10 µg/mL) increased the LDH release from damaged cancer cells by 37.6%, while similar results in LS180 cells were observed in response to Mp-CPS at a concentration of 100 µg/mL (increase by 33.1%). The most resistant to Mp-CPS were Caco-2 cells, wherein cell membrane damage was observed only after exposure to polysaccharides at the concentration of 100 µg/mL (increase by 10.8%). Studies conducted on human colon epithelial CCD841 CoN cells revealed no cytotoxic response to Mp-CPS, indicating the high selectivity of anticancer action of tested compounds. Moreover, the results of the LDH assay (Figure 2 and Table 4) revealed a positive correlation between the anticancer activity of Mp-CPS and colon cancer undifferentiation and invasiveness. The EC50 values (concentration causing cytotoxicity in 50% of cells compared to the control) calculated based on the results of the LDH assay were as follows: 2.673 × 10^21^ µg/mL (CCD841 CoN), 524 µg/mL (Caco-2), 371 µg/mL (LS180), and 174 µg/mL (HT-29). It is worth indicating that Mp-CPS had the worst efficiency in the elimination of Caco-2 cells, which are the least invasive but the most differentiated cell line among the tested ones. On the contrary, the most spectacular cytotoxic effect after Mp-CPS treatment was observed in HT-29 cells representing the advanced stage of colon cancer development [22,23].

**Figure 3:**
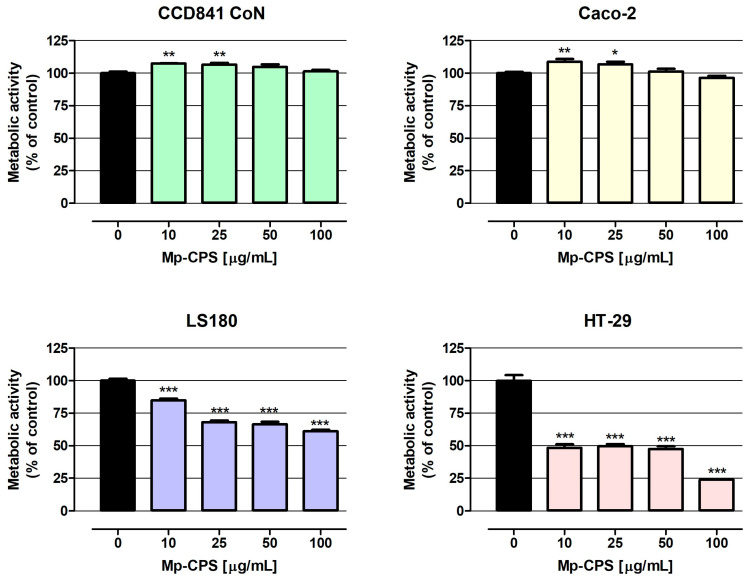
Antiproliferative effect of crude polysaccharides from *M. procera* on both normal and cancer colon cell lines. Human colon epithelial CCD841 CoN cells and human colon adenocarcinoma Caco-2, LS180, and HT-29 cells were treated with the crude polysaccharides Mp-CPS at concentrations of 10, 25, 50, and 100 μg/mL for 96 h. Mp-CPS’s impact on cell metabolic activity was measured photometrically by the MTT assay. Results are presented as mean ± SEM of at least 4 measurements. * *p* < 0.05 versus control, ** *p* < 0.01 versus control, *** *p* < 0.001 versus control, one-way ANOVA test; post-test: Dunnett.

The results of the MTT assay (Figure 3) show that both CCD841 CoN and Caco-2 cells treated with Mp-CPS at the concentrations of 10 and 25 µg/mL increased metabolic activity (on average by 7.1% and 7.9%, respectively), while polysaccharides at concentrations of 50 and 100 µg/mL did not impact the proliferation of indicated cells. On the contrary, Mp-CPS revealed a strong antiproliferative effect against LS180 and HT-29 cells. Similar to previously described data, the most significant changes were observed in HT-29 cells, wherein Mp-CPS decreased cell proliferation by 51.7% (10 µg/mL) to 76.2% (100 µg/mL). Proliferation of LS180 cells treated with Mp-CPS was reduced from 15.1% (10 µg/mL) to 38.9% (100 µg/mL). Discovered antiproliferative properties of Mp-CPS against LS180 and HT-29 cells were reflected in the IC50 values (concentration causing proliferation inhibition by 50% compared to the control) presented in Table 4. The IC50 values determined for LS180 and HT-29 cells based on the results of MTT assays were as follows: 232 µg/mL and 15 µg/mL. It should be highlighted that the MTT assay also proved the great selectivity of the anticancer action of tested compounds understood as the lack of a negative influence on the proliferation of the normal colon epithelium (CCD841 CoN) and the model of the intestinal epithelium (Caco-2). At the same time, the significant inhibition of cancer cell proliferation (LS180 and HT-29) in response to Mp-CPS was observed and detected that the antiproliferative effect intensified with the increase in cancer malignancy [22,23].

## 3. Discussion

The previously optimized method enabled the efficient isolation of the polysaccharide fraction Mp-CPS from *M. procera* fruiting bodies in the present study. The application of a preliminary treatment of the fungal raw material with a polar solvent such as ethanol enabled the removal of low-molecular-weight compounds, as indicated by the composition analysis (Table 1). Subsequently, hot water was employed as the extraction solvent for polysaccharides. Ultrasound-assisted extraction was selected as the technique of choice to ensure that the entire procedure complied with the principles of green chemistry while remaining environmentally safe and sustainable. As a result, we obtained Mp-CPS with a yield of 15.7% (Table 1), whereas other researchers reported yields of only 2.9% when using boiling water for extraction [14]. Chemical composition analysis of Mp-CPS revealed that it is composed mainly of sugars (63.86 ± 0.92%), which is consistent with data from other studies, where carbohydrates accounted for 74.1 ± 0.7% [14] and 69.37 ± 1.50% [5] of polysaccharide fraction. The main monosaccharide determined in Mp-CPS was glucose, which constituted nearly half of all sugars detected. Similarly to our study, Georgiev et al. revealed that glucose (62.3%) was the predominant component of the polysaccharide complex isolated from *M. procera*. They also reported a galactose content of approximately 20%, which is comparable to the values obtained in our study [14]. Deveci et al. reported glucose and galactose as the predominant monosaccharides in the polysaccharide extract of *M. procera*, though in different relative proportions. In their analysis, galactose constituted more than half of the total monosaccharide content, while glucose accounted for approximately one-fifth [5]. Fucose was identified as an additional constituent of Mp-CPS, and its content was determined to be comparable to values described in prior studies [5,14]. Wang et al. investigated polysaccharides obtained from *M. procera* mycelial cultures, identifying glucose and galactose as the major monosaccharides. In addition, they reported the presence of sugars such as mannose, ribose, rhamnose, xylose, arabinose, and fucose [17]. Some differences are likely attributable to the source of the fungal material, as distinct compounds may be synthesized in mycelial cultures compared to fruiting bodies harvested from natural habitats. To the best of our knowledge, trehalose and mannitol have not yet been reported in polysaccharides from *M. procera*, further highlighting the unique composition observed in our study. The observed heterogeneity in monosaccharide content may reflect a complex heteropolysaccharide structure in the studied mushroom, potentially underlying its notable biological properties.

Considering the glucan analysis of parasol mushroom, it was found that it contains predominantly β-glucans, while α-glucans represented only a minor fraction. Similar results were reported by Mirończuk-Chodakowska and Witkowska who reported that *M. procera* contains 11.4 ± 2.34 g/100 g DM of total glucans where β-glucans and α-glucans constituted 10.50 ± 0.30 g/100 g DM and 0.90 ± 0.58 g/100 g DM, respectively [24]. The remaining analyzed compound groups constituted only minor components of the polysaccharide fraction Mp-CPS. Application of the Sevag reagent resulted in a reduction in protein content to 4.01 ± 0.18%, in contrast to other studies where no deproteinization procedure was employed and protein levels reached 12.7 ± 0.2% [14]. Despite this treatment, a small fraction of protein remained, which may be explained by proteins being tightly bound to polysaccharide molecules or existing in complexes. The content of polyphenolic compounds was similar to that reported by Georgiev et al., amounting to approximately 2%, which can be considered a trace level [14]. Therefore, *M. procera* can be considered a rich source of polysaccharides, and appropriately selected extraction techniques allow their isolation in high yields and with satisfactory purity. This species is widely distributed in natural habitats, and reports on its successful cultivation further enhance its potential as a sustainable source of bioactive compounds [25]. Taken together, these characteristics highlight *M. procera* as a promising candidate for the development of functional foods, nutraceuticals, or other applications exploiting its biologically active polysaccharides. Previous studies have shown that *M. procera*, like many other wild edible mushrooms, may accumulate certain trace elements, particularly cadmium [26]. This feature is mainly influenced by the soil composition, pollution level, and geographical location of collection, and therefore, concentrations may vary widely depending on the site [27]. Furthermore, the extraction and purification procedures used to isolate polysaccharides may additionally reduce the risk of contamination. While the potential for heavy metal accumulation should be considered in the context of food safety, proper sourcing, environmental monitoring, and quality control can effectively minimize these risks, allowing *M. procera* to remain a valuable edible species and a promising source of bioactive compounds.

In the present study, we investigated three directions of biological activity of Mp-CPS, namely antioxidant, anti-inflammatory, and anticancer properties. Several studies demonstrated the great antioxidant properties of mushroom polysaccharides based on free radical scavenging, chelating Fe^2+^, or inhibition of lipid peroxidation [28]. Our study revealed that Mp-CPS exhibits antioxidant properties based on TEAC and ORAC assays. These findings are in line with those of Deveci et al., who investigated polysaccharide extracts from four edible mushroom species and reported that *M. procera* exhibited the highest antioxidant activity, as determined by DPPH^•^ scavenging, cupric reducing antioxidant capacity (CUPRAC), and phosphomolybdenum reducing antioxidant power (PRAP) assays [5]. In our study, antioxidant potential was also confirmed; however, the use of different analytical methods precludes direct numerical comparison of the results. As shown in Table 2, the antioxidant capacity of Mp-CPS determined using the ORAC assay was 358.56 ± 13.18 µM Trolox/g of Mp-CPS, which is comparable to the results reported by Georgiev et al. (313.3 ± 23.9 µM TE/g of polysaccharides) [14]. The antioxidant activity of the polysaccharide fraction Mp-CPS was approximately three times higher in the ORAC assay compared with the TEAC assay. This difference reflects the distinct mechanisms underlying these assays. ORAC primarily evaluates the ability of compounds to scavenge peroxyl radicals through hydrogen atom transfer (HAT), which closely mimics oxidative processes occurring in biological systems, such as lipid peroxidation. In contrast, TEAC measures the electron transfer (ET) capacity against the ABTS^•+^ radical cation, a non-physiological species, providing a more general indication of antioxidant potential [29]. The higher activity observed in ORAC suggests that the polysaccharide fraction is particularly effective at neutralizing peroxyl radicals via hydrogen donation, a mechanism highly relevant to cellular oxidative protection. This finding indicates a promising potential for the fraction to mitigate oxidative stress in vivo, supporting its relevance as a biologically active antioxidant. Overall, these results emphasize the significance of the hydrogen atom transfer mechanism in the antioxidant behavior of the tested polysaccharides and suggest that ORAC values may more accurately reflect their potential physiological impact.

Mushroom polysaccharides have been increasingly recognized for their immunomodulatory and anti-inflammatory properties. Numerous studies have demonstrated that these biopolymers can modulate signaling pathways involved in the inflammatory response, as well as inhibit the activity of pro-inflammatory enzymes and mediators. Such effects underline the potential of mushroom-derived polysaccharides as natural agents for the prevention and management of inflammation-related disorders [30]. Cyclooxygenases (COX-1 and COX-2) and lipoxygenases (LOX) are key enzymes in the metabolism of arachidonic acid, leading to the production of pro-inflammatory mediators that play a central role in the initiation and progression of inflammation. Inhibition of these enzymes by natural compounds is considered beneficial for health, as it may help prevent or mitigate chronic inflammation and its associated disorders, including inflammatory bowel diseases and colorectal cancer [31,32]. The tested Mp-CPS fraction inhibited the activity of all investigated pro-inflammatory enzymes, namely COX-1, COX-2, and LOX. The most pronounced effect was observed against COX-1, whereas the inhibition of COX-2 and LOX was weaker and of similar magnitude. When compared with acetylsalicylic acid (ASA), Mp-CPS proved to be a less potent inhibitor overall; however, the inhibition level of approximately 40% still represents a considerable activity in the context of natural extracts. Comparable anti-inflammatory effects of mushroom-derived polysaccharides have been reported in the literature; however, to date, the direct effects of polysaccharides from *M. procera* on COX and LOX enzymes have not been investigated. Polysaccharides from several fungal species, e.g., *Hericium erinaceus*, *Panaeolus cyanescens*, *Armillaria mellea*, and *Sparassis crispa*, have been shown to suppress COX and LOX expression and activity [20,33,34]. In reference to our previous studies on polysaccharides from edible macrofungi, it can be observed that *M. procera* exhibits the highest activity against LOX; for instance, *S. crispa* inhibited this enzyme by 23.8% [20]. In contrast, *A. mellea* showed no activity toward COX-2, whereas Mp-CPS caused its inhibition by 39.09 ± 0.42%, highlighting the relatively strong and broad inhibitory potential of this species compared to other mushroom-derived polysaccharides.

Colorectal cancer is one of the most prevalent and deadly malignancies worldwide; at the same time, its strong dependence on the diet indicates food components as natural preventive agents. Edible mushrooms with proven health-promoting properties are emerging as promising candidates for testing their chemopreventive potential in the context of colorectal cancer. Next to the diverse array of bioactive compounds present within them, among which polysaccharides are well-described anticancer agents [35,36,37], they also attract scientists’ attention because their use in cancer prevention seems to be safe and affordable [38]. In this light *M. procera* deserves consideration, especially that the anticancer abilities of this mushroom have been previously reported [18,39,40,41]. Nevertheless, the indicated research concern only extracts prepared with organic solvents such as methanol or ethanol [18,39,40,41]. Studies performed by Arora et al. focused on the anticancer effects of both water and ethanol extracts, showing their great cytotoxic and antiproliferative influence in three human cancer cell lines ACHN (kidney), MCF-7 (breast), and COLO-205 (colon) in response to 48 h of treatment. In the case of colon cancer cells, the IC50 values appointed based on the results of the Trypan Blue Viability Test were as follows: 349.1 µg/mL (aqueous extract) and 159.4 µg/mL (ethanolic extract) [39]. Similarly, studies conducted by Kosanic et al. on three other human cancer cell lines A549 (lung), HeLa (cervical), and LS174 (colon) also proved the antiproliferative effect of *M. procera* methanol extract. Based on the results of the MTT assay performed after 72 h of LS174 cells treatment, the IC50 value was determined as 68.49 µg/mL [41]. What is interesting is that other studies on the anticancer activity of methanol extract from *M. procera* performed by Ćirić et al. revealed the lack of its beneficial influence on the following human cancer cell lines: MCF-7 (breast), NCI-H460 (lung), HeLa (cervical), and HepG2 (liver) [42]. There are also two other scientific reports strictly focused on the elimination of human lung adenocarcinoma A549 cells by *M. procera* extracts, among which the first showed the great anticancer potential of ethanol extract [18], while the second one demonstrated the weak antiproliferative abilities of the tested extract [40].

Despite the anticancer abilities of *M. procera* extracts being demonstrated, there is still a lack of studies evaluating the possibility of using its polysaccharides in the prevention and treatment of colon cancer. The presented study was the answer to this knowledge gap. Using the panel of human colon cancer cell lines, representing the successive stages of disease development based on Dukes classification (Caco-2: Dukes B, LS180: Dukes B, HT-29: Dukes C) as well as human normal colon epithelial CCD 841 CoN cells, our research revealed the great chemopreventive properties of the investigated polysaccharides Mp-CPS. The tested compounds revealed strong cytotoxic abilities in Caco-2, LS180, and HT-29 cells (EC50 values calculated based on LDH assay were 524 µg/mL, 371 µg/mL, and 174 µg/mL, respectively), as well as the strong antiproliferative capacity in LS180 and HT-29 cells (IC50 values calculated based on MTT assay were 232 µg/mL and 15 µg/mL, respectively). At the same time Mp-CPS did not negatively impact on the viability and proliferation of normal colon epithelial cells. Cited data clearly demonstrated the significant selectivity of tested compounds’ action—no adverse effects in normal cells, and the effective elimination of cancer cells which intensified with the degree of tumor malignancy. Our study corresponds with the data obtained by Deveci et al., who using Alamar blue assay revealed the inhibition of HT-29 cells’ growth, reaching 95% after 18 h of incubation with *M. procera* polysaccharide extract at the concentration of 500 µg/mL [5]. It is also worth mentioning that Deveci et al. revealed the strong anticancer properties of *M. procera* polysaccharide extract in hepatoma (HepG2) and cervical carcinoma (HeLa) cell lines; however, the most significant changes they recorded were in the mentioned HT-29 cells [5]. Unfortunately, according to our best knowledge, there is not another research focused on the anticancer properties of *M. procera* polysaccharides. Thus, the presented results are unique and worth sharing. An additional advantage of our research, despite its preliminary nature, is the inclusion of normal cells in the analysis, which, surprisingly, is rarely performed.

## 4. Materials and Methods

### 4.1. Materials

*Macrolepiota procera* (Scop.) Singer fruiting bodies were collected from the natural state in Majdan Nowy (Lubelskie region, Poland; GPS: 50°28′12.027″ N; 22°43′55.009″ E) in August 2022. Mushroom samples were verified by the authors and authenticated by Prof. Renata Nowak from the Department of Pharmaceutical Botany, Medical University of Lublin, Poland. Voucher specimen (No. MSH-045) was deposited at the Department of Pharmaceutical Botany, Medical University of Lublin, Poland. The harvested mushrooms were freeze-dried, ground into a fine powder, and stored at −30 °C in a freezer until analysis.

### 4.2. Chemicals

The COX (ovine) Colorimetric Inhibitor Screening Assay Kit was purchased from Cayman Chemical Company, Ann Arbor, MI, USA. The Megazyme Mushroom and Yeast Beta-Glucan Assay Kit was obtained from Megazyme International Ireland Ltd., Wicklow, UK. Redistilled phenol, Bradford reagent, bovine serum albumin (BSA), 2,20-azinobis-(3-ethylbenzothiazoline-6-sulfonic acid) (ABTS^•+^), Trolox, 2,2′-azobis (2-methylpropionamide) dihydrochloride (AAPH), gallic and linoleic acids, and soybean 15-lipoxygenase were provided by Sigma-Aldrich Chemical Co. (St. Louis, MO, USA). Fluorescein sodium salt was purchased from Roth (Karlsruhe, Germany). Di-sodium hydrogen phosphate dihydrate was purchased from Chempur (Piekary Slaskie, Poland). The standard of myo-inositol was purchased from Sigma-Aldrich Fine Chemical Co. (St. Louis, MO, USA). Mannitol, xylitol, sucrose, trehalose, fucose, galactose, glucose, mannose, ribose, and arabinose were provided by Dr. Ehrenstorfer (Augsburg, Germany). Milli-Q quality (Millipore, Bedford, MA, USA) water was used throughout the HPCE analysis. Sodium hydroxide solution 1 M for HPCE was purchased from Merck (Darmstadt, Germany). The in vitro Toxicology Assay Kit Lactate Dehydrogenase Based, thiazolyl blue tetrazolium bromide (MTT), Dulbecco’s modified Eagle’s medium (DMEM), DMEM/Nutrient Mixture F-12 Ham (DMEM/F-12 Ham), fetal bovine serum (FBS), penicillin, and streptomycin were obtained from Sigma Aldrich (St. Louis, MO, USA). Eagle’s Minimum Essential Medium (EMEM) was obtained from the American Type Culture Collection (ATCC, Manassas, VA, USA). All other chemicals and solvents were of analytical grade and were purchased from Avantor Performance Materials Poland (Gliwice, Poland).

### 4.3. Preparation of Crude Polysaccharides from M. procera Mp-CPS

Mp-CPS was extracted during the two-step procedure involving alcohol and water extraction described in detail in a previous paper [20]. Dried mushroom material (100 g) was macerated with ethanol (1:5 g/mL) for 24 h prior to the ultrasound-assisted extraction (UAE) with alcohol (1:5 g/mL) performed for 30 min at room temperature. Then the air-dried residue was subjected to doubled UAE with hot water (1:10 g/mL) for 30 min at 90 °C. Proteins were removed from the aqueous extract by Sevage reagent (chloroform/isoamyl alcohol, 4:1, *v*/*v*). Finally, the Mp-CPS was precipitated with cold anhydrous ethanol (1:4, *v*/*v*) overnight at 4 °C, centrifuged (8000 rpm, 5 min), and lyophilized before further analysis.

### 4.4. Determination of the Total Sugar Content

The total sugar content in the Mp-CPS was evaluated according to the method described by Dubois et al. [43]. The results were calculated into glucose equivalents and expressed as % of Mp-CPS.

### 4.5. Determination of Uronic Acids

The total content of uronic acids in the Mp-CPS was determined using the method given in detail by He et al. [44]. The results were calculated into galacturonic acid equivalents and expressed as % of Mp-CPS.

### 4.6. Determination of Proteins

The total content of protein in the Mp-CPS was established with the Bradford method using bovine serum albumin (BSA) as a standard compound [45]. The results were expressed as % of Mp-CPS.

### 4.7. Determination of the Total Content of Phenolic Compounds

The content of phenolics was investigated using the protocol described in detail by Olech et al. [46]. Briefly, 20 µL of the Mp-CPS solution was combined with 20 µL of Folin–Ciocalteu reagent (diluted 1:4, *v*/*v* with distilled water), after which 160 µL of sodium carbonate solution (75 g/L) was added. The mixture was incubated for 20 min, and absorbance was subsequently measured at 680 nm using a microplate reader. The results were calculated into gallic acid equivalents and expressed as % of Mp-CPS.

### 4.8. Monosaccharide Composition of the Mp-CPS

Monosaccharide composition of the Mp-CPS was determined after hydrolysis of 100 mg of the sample with 2 M trifluoroacetic acid (TFA) at 100 °C for 2 h. TFA was removed under reduced pressure, and the hydrolysate was dissolved in water and centrifuged (10,000 rpm, 5 min). The carbohydrate profile was analyzed by high-performance capillary electrophoresis (HPCE) using a PA 800 plus system (Sciex, Framingham, MA, USA) with a photodiode array detector (λ = 270 nm), following a modified method of Rovio et al. [47]. Separation was performed in a bare fused silica capillary (25 µm i.d., 60.2 cm total length, 50.0 cm effective length) at 15 °C, under 30 kV. Samples were introduced by pressure injection (1.0 psi, 15 s) followed by a short water plug (0.5 psi, 10 s). Data were processed with 32 Karat Software (v9.1), and monosaccharides were identified by comparison of migration times with standards using the standard addition method.

### 4.9. Evaluation of the Total α- and β-Glucans Content

The contents of total glucans, as well as α- and β-glucans, in fruiting bodies of *M. procera* were determined using the Megazyme Mushroom and Yeast Beta-Glucan Assay Kit, according to the manufacturer’s protocol presented in detail previously in [20]. Results were expressed as g/100 g of dry mushroom material.

### 4.10. Determination of Biological Activity of the Mp-CPS

#### 4.10.1. TEAC (Trolox Equivalent Antioxidant Capacity) Assay

The antioxidant properties of Mp-CPS were evaluated using the Trolox Equivalent Antioxidant Capacity (TEAC) assay, as previously described [48]. Briefly, an ABTS^•+^ (2,2′-azino-bis(3-ethylbenzothiazoline-6-sulfonic acid)) solution (0.096 mg/mL in methanol) was mixed with the sample and incubated at 30 °C for 6 min. The absorbance was then measured at 734 nm. Results were expressed as μM of Trolox per gram of crude polysaccharides.

#### 4.10.2. ORAC (Oxygen Radical Absorbance Capacity) Assay

The ORAC assay was performed according to the method of Dienaitė et al. [49], with modifications introduced by Olech et al. [50]. The reaction mixture contained the tested sample and fluorescein solution (10 nM). After incubation for 20 min at 37 °C, AAPH (240 mM) was added. Fluorescence was then measured at 485 nm (excitation) and 515 nm (emission). Results were expressed as μM of Trolox per gram of crude polysaccharides.

#### 4.10.3. Inhibition of Cyclooxygenase (COX) Activity

The ability of the Mp-CPS sample (0.5 mg/mL) to inhibit two isoforms of cyclooxygenase, COX-1 and COX-2, was evaluated using the COX (ovine) Colorimetric Inhibitor Screening Assay Kit, according to the manufacturer’s instructions. The detailed procedure was described previously by Łubek-Nguyen et al. [48].

#### 4.10.4. Inhibition of Lipoxygenase (LOX) Activity

The inhibitory effect of Mp-CPS on 15-lipoxygenase activity was evaluated according to the method of Maiga et al. [51]. Samples (5 mg/mL) were mixed with 0.2 M borate buffer (pH 9.0), 15-lipoxygenase (167 U/mL), and linoleic acid (134 μM). Absorbance was measured at 234 nm. Acetylsalicylic acid (10 mM) was used as the reference inhibitor. The percentage of 15-lipoxygenase inhibition was calculated relative to the control sample (100% activity) without Mp-CPS.

#### 4.10.5. Anticancer Potential—In Vitro Studies

The human colon epithelial cell line CCD841 CoN and the human colon adenocarcinoma cell line Caco-2 were obtained from the American Type Culture Collection (ATCC, Manassas, VA, USA). The human colon adenocarcinoma cell lines LS180 and HT-29 were purchased from the European Collection of Cell Cultures (ECACC, Centre for Applied Microbiology and Research, Salisbury, UK). The indicated cell lines were cultivated under conditions consistent with the guidelines of the collections from which they were purchased.

To determine the antiproliferative effect of Mp-CPS, cells were seeded on 96-well microplates at a density of 3 × 10^4^ cells/mL (cancer cells) and 5 × 10^4^ cells/mL (normal cells), while to investigate polysaccharides’ cytotoxicity, cells were plated at a density of 5 × 10^4^ cells/mL (cancer cells) and 1 × 10^5^ cells/mL (normal cells). The next day, the culture medium was removed, and the cells were treated with Mp-CPS at concentrations of 10, 25, 50, and 100 µg/mL. Dilutions of Mp-CPS were prepared in the fresh medium with 10% FBS (MTT test) or 5% FBS (LDH test). The cell proliferation in response to Mp-CPS was assessed after 96 h using the MTT assay, while cytotoxicity was examined after 24 h of cell exposure to Mp-CPS using the LDH assay. The description of the execution of the indicated tests was previously presented [20].

### 4.11. Statistical Analysis

All results were expressed as the mean ± standard deviation (SD) from three replications. Calculations were performed in STATISTICA 10.0 (StatSoft Poland, Cracow, Poland). The data from the anticancer activity determination were presented as the mean value and standard error of the mean (SEM). Statistical analysis was performed using one way-ANOVA with the Dunnett post-test and column statistics used for comparisons. Significance was accepted at *p* < 0.05. The IC50 value (half-maximal inhibitory concentration) and EC50 value (concentration causing cytotoxicity in 50% of cells compared to the control) were calculated using GraphPad Prizm 5.

## 5. Conclusions

In the present study, the previously optimized ultrasound-assisted extraction method was employed to obtain a polysaccharide-rich fraction Mp-CPS from the fruiting bodies of *M. procera*. Detailed compositional analysis revealed that Mp-CPS is primarily composed of glucose- and galactose-based heteropolysaccharides, with β-glucans as the dominant glucan type. Biological activity assessment showed that Mp-CPS possesses strong antioxidant potential, particularly in ORAC assays, suggesting effective hydrogen atom transfer-based radical scavenging relevant to cellular protection against oxidative stress. Moreover, Mp-CPS displayed noteworthy anti-inflammatory activity, inhibiting key enzymes of arachidonic acid metabolism (COX-1, COX-2, and LOX), thereby confirming its potential role in the prevention of inflammation-related disorders. Furthermore, the performed study revealed the great chemopreventive properties of Mp-CPS, which effectively eliminated various colon cancer cells, without any cytotoxic effects on normal colon epithelial cells. The discovered anticancer abilities of MP-CPS that intensified with the degree of cell malignancy, together with the lack of negative influence on normal epithelium, indicates both high selectivity and safety for their further therapeutic applications. Importantly, this is the first report indicating anticancer activity of polysaccharides derived from *M. procera* against colorectal cancer cells in vitro, broadening the scope of known bioactivities of this species.

Taken together, these findings highlight *M. procera* as a sustainable and promising source of biologically active polysaccharides with antioxidant, anti-inflammatory, and anticancer potential. The unique chemical profile and broad spectrum of activities of Mp-CPS suggest its applicability in the development of functional foods and nutraceuticals. Future studies should further elucidate the molecular mechanisms of action and evaluate the in vivo efficacy of *M. procera* polysaccharides to fully explore their biomedical potential. Additionally, comprehensive structural characterization of the individual polysaccharide components within the Mp-CPS fraction and investigation of their specific contribution to the observed bioactivities will be essential for advancing their practical applications.

## Figures and Tables

**Table 1:** The chemical composition of crude polysaccharides from *M. procera* Mp-CPS; the contents of total glucans, α-glucans and β-glucans in the fruiting bodies of *M. procera* presented as g/100 g of dry weight and yield of Mp-CPS extraction. Data are presented as mean values ± standard deviation from three replicate determinations.

Group of Compounds	Result (Mean Value ± SD)
Sugars (% of Mp-CPS)	63.86 ± 0.92
Uronic acids (% of Mp-CPS)	6.71 ± 0.21
Proteins (% of Mp-CPS)	4.01 ± 0.18
Phenolic compounds (% of Mp-CPS)	2.19 ± 0.09
Total glucans (g/100 g of d.w.)	13.51 ± 0.41
α-glucans (g/100 g of d.w.)	2.75 ± 0.13
β-glucans (g/100 g of d.w.)	10.76 ± 0.53
Yield (%)	15.7 ± 0.38

**Table 2:** Antioxidant activity of crude polysaccharides from *M. procera* Mp-CPS determined by the TEAC assay (Trolox Equivalent Antioxidant Capacity) and ORAC assay (Oxygen Radical Absorbance Capacity). Data are presented as mean values ± standard deviation from three replicate determinations.

Antioxidant Test	Result (Mean Value ± SD)
TEAC (µM Trolox/g of Mp-CPS)	102.00 ± 2.09
ORAC (µM Trolox/g of Mp-CPS)	358.56 ± 13.18

**Table 3:** Inhibitory activity of crude polysaccharides from *M. procera* Mp-CPS against pro-inflammatory enzymes: cyclooxygenase-1 (COX-1), cyclooxygenase-2 (COX-2), and lipoxygenase (LOX) expressed in %. Acetylsalicylic acid (10 mM) was used as a control. Data are presented as mean values ± standard deviation from three replicate determinations.

	COX-1	COX-2	LOX
	(% of Inhibition)		
Mp-CPS	74.23 ± 1.26	39.09 ± 0.42	43.69 ± 0.94
ASA	40.21 ± 0.78	96.84 ± 0.93	92.86 ± 0.51

**Table 4:** The EC50 value (concentration causing cytotoxicity in 50% of cells compared to the control) and IC50 value (concentration causing proliferation inhibition by 50% compared to the control) determined for normal and cancer cell lines based on the results of LDH and MTT assays, respectively.

	Assay	LDH Assay		MTT Assay	
Cell Line		EC50 [µg/mL]	Trust Range [µg/mL]	IC50 [µg/mL]	Trust Range [µg/mL]
CCD841 CoN		2.67 × 10^21^	no data	no data	no data
Caco-2		524	141–1949	no data	no data
LS180		371	166–828	232	137–394
HT-29		174	48–631	15	8–29

## Data Availability

Detailed information is available upon request from the corresponding author.
